# Regulation of neurotrophin receptor (Trk) signaling: suppressor of cytokine signaling 2 (SOCS2) is a new player

**DOI:** 10.3389/fnmol.2014.00039

**Published:** 2014-05-14

**Authors:** Rachel T. Uren, Ann M. Turnley

**Affiliations:** Neural Regeneration Laboratory, Centre for Neuroscience Research and Department of Anatomy and Neuroscience, The University of MelbourneMelbourne, VIC, Australia

**Keywords:** NGF, ubiquitin, signal transduction, Trk receptors, PC12 cells, DRG neurons, SOCS-2, neurite outgrowth

## Abstract

The classic neurotrophins Nerve Growth Factor (NGF), Brain Derived Neurotrophic Factor (BDNF) and Neurotrophins NT-3 and NT-4 are well known to regulate various aspects of neuronal differentiation, survival and growth. They do this by binding to their cognate receptors, members of the Tropomyosin-related kinase (Trk) receptor tyrosine kinase family, namely TrkA, TrkB, and TrkC. These receptors are then internalized and localized to different cellular compartments, where signal transduction occurs. Conversely, members of the suppressor of cytokine signaling (SOCS) family are best known as negative regulators of signaling via the JAK/STAT pathway. Some members of the family, and in particular SOCS2, have roles in the nervous system that at least partially overlap with that of neurotrophins, namely neuronal differentiation and neurite outgrowth. Recent evidence suggests that SOCS2 is a novel regulator of NGF signaling, altering TrkA cellular localization and downstream signaling to affect neurite growth but not neuronal survival. This review first discusses regulation of Trk receptor signaling, followed by the role of SOCS2 in the nervous system and finishes with a discussion of possible mechanisms by which SOCS2 may regulate TrkA function.

## Introduction

A great deal of interest has focused on the therapeutic potential of neurotrophins to prevent neurodegeneration and promote neural regeneration in a range of neurological diseases and neurotrauma (Allen et al., 2011; Colafrancesco and Villoslada, 2011; Ossipov, 2011; Duman and Voleti, 2012). Although administration of neurotrophins, such as NGF, has promoted improvement in many studies, their use as a therapeutic option is limited by undesirable side-effects, such as neuropathic pain. Therefore, other approaches to target neurotrophin receptors is required for therapeutic purposes, to modulate specific aspects of neurotrophin signaling that are beneficial and avoid those that are detrimental. To achieve this, a deeper understanding of the mechanisms that regulate neurotrophin receptor function and signaling is required. Here we describe the potential role of SOCS2 as a novel regulator of NGF receptor (TrkA) signaling that differentially affects NGF-mediated neurite outgrowth but not neuron survival, altering TrkA protein levels, cellular localization and extent and duration of specific NGF-induced signal transduction pathways.

## The classical neurotrophins and their receptors

The presence of a soluble factor capable of supporting the survival of motor and sensory neurons was first demonstrated in 1949 and prompted the characterization of NGF (Hamburger and Levi-Montalcini, 1949; Levi-Montalcini, 1987), one of the earliest identified growth factors and the first member of the “classical neurotrophin” family. In addition to its role in the peripheral nervous system, NGF has been shown to have actions in the central nervous system and the immune system (Dreyfus, 1989; Dreyfus et al., 1989; Snider, 1994; Ernsberger, 2009; Scuri et al., 2010; Allard et al., 2012). Three additional mammalian neurotrophic factors have since been identified; brain-derived neurotrophic factor (BDNF), neurotrophin-3 (NT-3) and neurotrophin-4/5 (NT-4) (reviewed by (Reichardt, 2006; Skaper, 2012).

Neurotrophins signal through two-types of cell surface receptor; the p75 neurotrophin receptor (p75^NTR^) (Radeke et al., 1987; Bibel et al., 1999) and the Tropomyosin-related kinase (Trk) receptor tyrosine kinases (Kaplan et al., 1991a,b; Klein et al., 1991a). They bind with equal affinity to p75^NTR^ (Radeke et al., 1987; Rodriguez-Tebar et al., 1990; Hallbook et al., 1991; Rodriguez-Tebar et al., 1992), however, the neurotrophins are more selective in their interactions with the Trk receptors; TrkA binds NGF, TrkB binds BDNF and NT-4/5 and TrkC binds NT-3 (Kaplan et al., 1991a,b; Klein et al., 1991a; Dechant et al., 1993; Mahadeo et al., 1994). The mature neurotrophins form homodimers that can simultaneously bind two receptor molecules (Barbacid et al., 1991; Heymach and Shooter, 1995). The Trk receptors can form homodimers or can associate with p75^NTR^ (Bibel et al., 1999), thus a single neurotrophin dimer can simultaneously bind a Trk:Trk or Trk:p75^NTR^ complex. When the Trks form a co-receptor complex with p75^NTR^, lower concentrations of the neurotrophic ligand are required to initiate signaling via the Trk receptor than if the Trk receptors were solely expressed (Mahadeo et al., 1994). The co-receptor Sortilin can also bind the Trk receptors and has been implicated in the transport of TrkA along axons (Vaegter et al., 2011). Beyond its involvement in Trk and sortilin signaling, p75^NTR^ has been implicated in the inhibition of axon growth and dendritic complexity by regulating the GTPase RhoA (Yamashita et al., 1999, 2002; Zagrebelsky et al., 2005).

### Signaling events downstream of the Trk neurotrophin receptors

**Figure 1:**
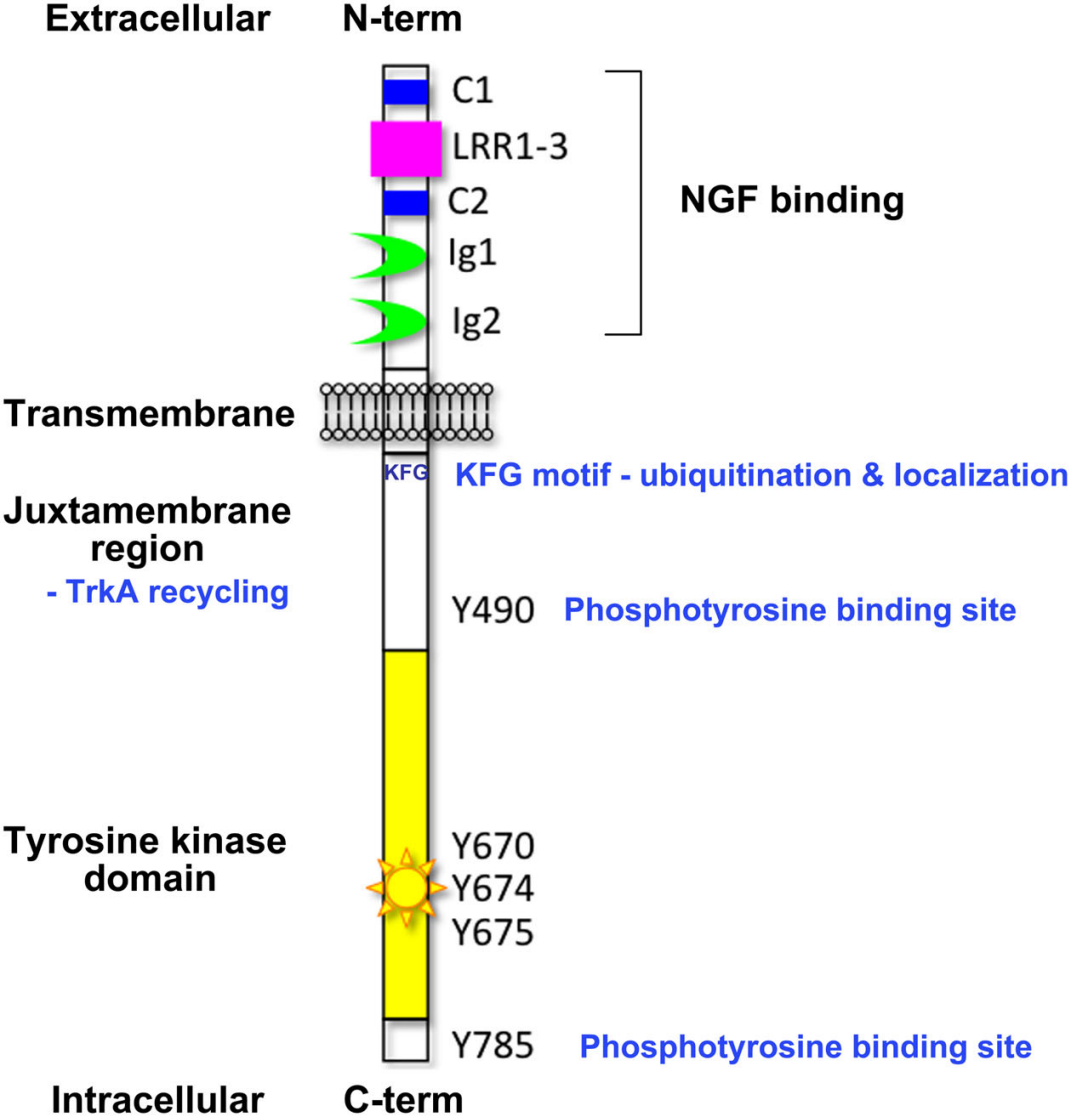
**Domain organization of the Trk neurotrophin receptors**. This figure highlights the key structural elements of the Trk neurotrophin receptors; the extracellular ligand-binding domain comprising leucine-rich motifs (LRR1-3), two cysteine clusters (C1 and C2) and two immunoglobulin-like domains (Ig1 and Ig2), the transmembrane domain, and the intracellular tyrosine kinase domain. Critical tyrosine residues of the human TrkA intracellular domain are highlighted. Tyrosine residues at positions 670, 674, and 675 are located in the activation loop of the human TrkA tyrosine kinase domain. Tyrosine 490 forms a PTB binding motif (NPXpY) known to bind SHC and FRS2 (Obermeier et al., 1993b; Ong et al., 2000) and tyrosine 785 binds to PLCγ (Obermeier et al., 1993a). The juxtamembrane KFG motif is an ubiquitination site involved in receptor localization.

A diverse range of cell responses can be initiated upon neurotrophin binding. The type of signal is influenced by the combination of neurotrophin receptors presented on the cell membrane and the unique collection of intracellular effectors that can be recruited by the different receptors upon activation (Lee et al., 2001; Huang and Reichardt, 2003). Signaling downstream of the Trk receptors is a complex affair (Reichardt, 2006). The Trk receptors possess a single transmembrane domain and an extracellular ligand-binding domain comprising leucine-rich motifs (LRR1-3), two cysteine clusters (C1 and C2) and two immunoglobulin-like domains (Ig1 and Ig2) (Johnson et al., 1986; Radeke et al., 1987; Schneider and Schweiger, 1991; Windisch et al., 1995a,b) (Figure 1). The second immunoglobulin-like domain largely dictates the neurotrophin-binding specificity of the Trk receptors and there is some evidence that the immunoglobulin-like domains serve to inhibit spontaneous dimerization of the Trk receptors in the absence of ligand (Arevalo et al., 2000). Ligand binding to the Trk receptors triggers receptor dimerization and activation of the intrinsic cytoplasmic kinase domain and receptor autophosphorylation. There are 10 known conserved tyrosine residues on the cytoplasmic tail of the mammalian Trk receptors, some of which are phosphorylated upon receptor activation (Stephens et al., 1994; Inagaki et al., 1995). Three of these sites are located in the auto-regulatory loop of the tyrosine kinase domain and thus modulate kinase activity when phosphorylated. The remaining phosphorylated tyrosine residues form binding sites for scaffold proteins and enzymes that contain phosphotyrosine binding (PTB) domains or Src-homology-2 (SH2) domains and the specificity of these interactions is tailored by the flanking amino acids. The best characterized of these docking sites are Y490 and Y785 as per the nomenclature of human TrkA, also known as Y499 and Y794 in rat TrkA. The other conserved sites have been shown to display some degree of functional redundancy with respect to downstream signaling.

#### Activation of signaling pathways

**Figure 2:**
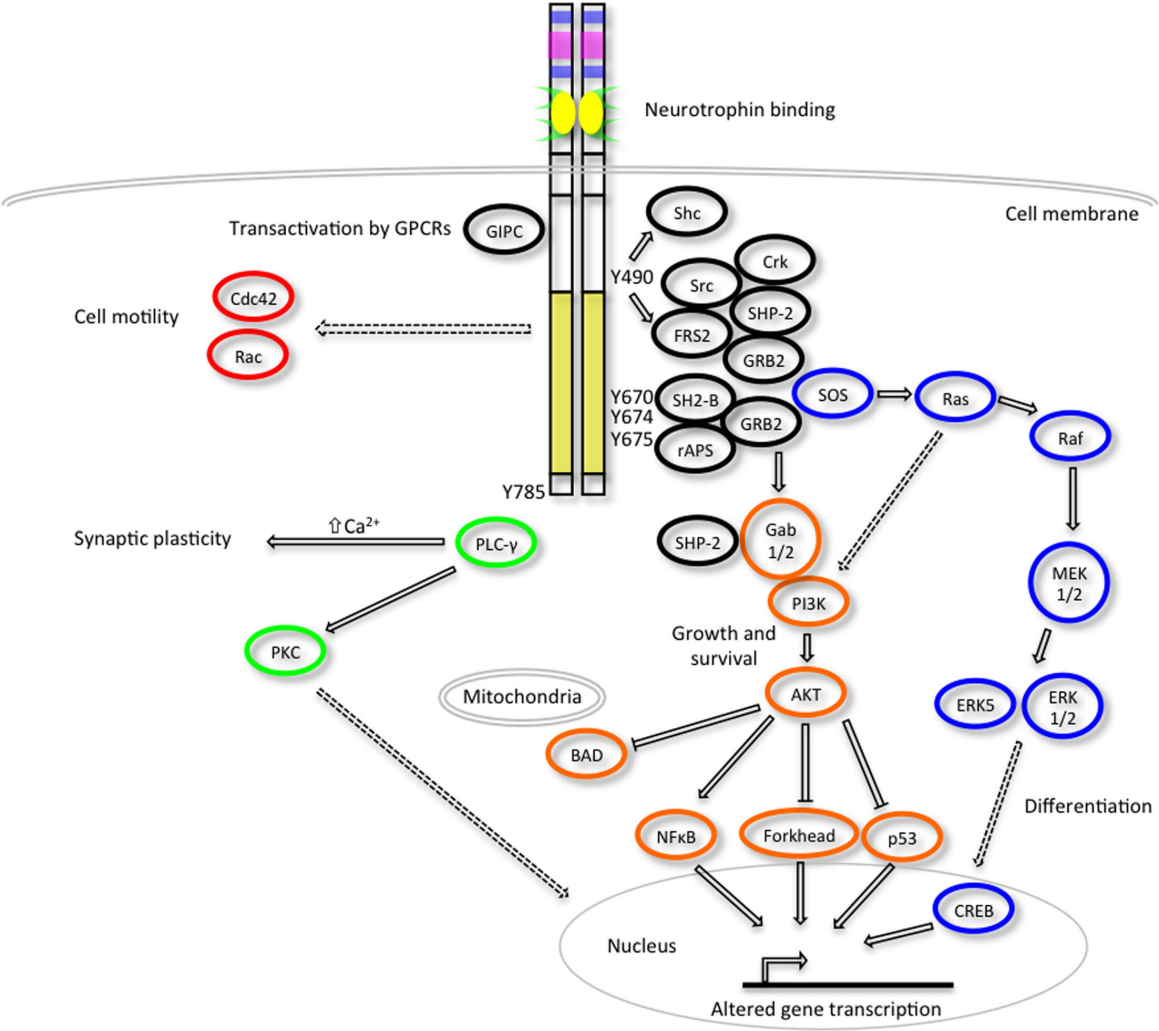
**Trk receptor signaling pathways**. Upon neurotrophin binding, the activated Trk receptors engage various intracellular signaling pathways such as those mediated by extracellular signal-regulated kinases (ERKs), phosphatidylinositol 3-kinase (PI3K) and phospholipase Cγ (PLC-γ) to promote cellular differentiation, growth, survival, synaptic plasticity and changes to cell motility (adapted from Huang and Reichardt, 2003). Critical tyrosine (Y) residues of the human TrkA intracellular domain are shown. Direct, indirect and inhibitory pathways are denoted by solid arrows, dashed arrows and blunt arrows respectively.

The activated Trk receptors initiate diverse signaling pathways downstream of Ras, phosphatidylinositol 3-kinase (PI3-kinase) and phospholipase C- γ1 (PLC-γ1) (Figure 2) (Obermeier et al., 1993a,b; Ong et al., 2000). These signaling components are common to many other growth factor and cytokine stimuli, thus there is obviously scope for crosstalk between these diverse signaling pathways. Both the Ras-MAPK and PI3-K/AKT pathways converge on the common upstream scaffold protein, Src homologous and collagen-like (Shc), which is known to recognize phosphorylated Y490 of the Trk receptors. This tyrosine residue is also well characterized as a FRS2 binding site which can also promote the prolonged activation of MAP kinases via recruitment of GRB2, Crk and Src. PLC γ1 binds the activated TrkA receptor at Y785 and prompts increased Ca^2+^ levels which can influence synaptic plasticity and activation of protein kinase C (PKC) signaling cascades to effect changes in transcription. The neurotrophins are also known to activate members of the Rho GTPase family such as Cdc42 and Rac that are regulators of the cytoskeletal rearrangements required for cell motility and the formation of growth cones (Yuan et al., 2003).

A less established pathway downstream of the Trk receptors is the activation of the signal transducer and activator of transcription-3 (STAT3) transcription factor. Early studies demonstrated that dominant negative STAT3 can promote PC12 neurite outgrowth in the absence of NGF (Ihara et al., 1997) and STAT3 was identified as a downstream effector of Trk neurotrophins in both PC12 cells and primary hippocampal neurons (Ng et al., 2006). BDNF can induce proliferation of neural stem cells via a mechanism involving MAP kinase, AKT and STAT3 activation (Islam et al., 2009) and constitutively active Trk receptors can induce STAT3 activation (Miranda et al., 2010).

A variety of phosphatases are recruited to the Trk receptors. The protein tyrosine phosphatase SH2 domain-containing phosphatase -2 (SHP-2) has been shown to have a transient association with the Trk receptors upon neurotrophin binding (Okada et al., 1996; Goldsmith and Koizumi, 1997) and provide an important counterbalance to TrkB activation (Rusanescu et al., 2005). SHP-2 may also be indirectly recruited to the Trk receptors via adapter molecules such as FRS2 (Yamamoto et al., 2005; Easton et al., 2006) and NOMA-GAP (Rosario et al., 2007). The related SHP-1 phosphatase has also been shown to bind TrkA at Y490 and dephosphorylate residues Y674 and Y675 in the TrkA tyrosine kinase activation loop (Marsh et al., 2003). The receptor-like protein tyrosine phosphatase sigma (PTPsigma) can bind stably to TrkA and TrkC via its transmembrane domain and dephosphorylate all the Trk receptors upon neurotrophin stimulation (Faux et al., 2007). Another receptor-like phosphatase, the protein tyrosine phosphatase receptor zeta (PTPRz), can inhibit phosphorylation of the activation loop tyrosine residues of the TrkA receptor upon NGF stimulation (Shintani and Noda, 2008). Interestingly, some phosphatases have a positive regulatory role in TrkA signal transduction. The protein phosphatase 2A has been shown to enhance the tyrosine kinase activity of the TrkA receptor leading to sustained activation and downstream AKT and ERK1/2 activity, possibly mediated by dephosphorylation of inhibitory serine and threonine phosphorylation sites (Van Kanegan and Strack, 2009).

The activated Trk receptors can modulate the signaling of various ion channels and, even in the absence of neurotrophins, the Trk receptors can be transactivated upon association with membrane associated G-protein coupled receptors (GPCRs) (Chao, 2003). The juxtamembrane domain of TrkA has been shown to interact with a PDZ domain-containing scaffold protein GIPC that is also a component of a GPCR complex (Lou et al., 2001). More recent studies have shown that GIPC is required for the efficient trafficking and endocytosis of TrkA (Varsano et al., 2006). This finding points to the critical role of receptor trafficking in the modulation of Trk-mediated signaling.

### Neurotrophin receptor dynamics and the unique cell biology of neurotrophin signaling

The retrograde transport of neurotrophic stimuli has been most extensively studied in the developing peripheral nervous system and is critical for the formation and refinement of neuronal circuits during development and for the regulation of synaptic plasticity and neuronal survival in the mature nervous system (Huang and Reichardt, 2001; Zweifel et al., 2005). The mechanics of this receptor trafficking have been scrutinized since retrograde axonal transport of NGF was first demonstrated in 1974 (Hendry et al., 1974; Paravicini et al., 1975; Johnson and Yip, 1985). Target tissues secrete limiting amounts of neurotrophins and neighboring nerve terminals will transport this trophic signal to the distant neuronal cell bodies and thus support the survival of the innervating neuron. The complex morphology of neurons poses specific challenges for the transport of molecules from one part of the cell to another, as axon lengths can be in excess of 1 meter in some instances. Passive diffusion cannot account for the speed and distance travelled by these signals, and several different mechanisms have been proposed for the propagation of NGF signals, however, a mounting body of evidence now supports a model of the active retrograde transport of the neurotrophin signal in neurons via “signaling endosomes” (reviewed by Howe and Mobley, 2005; Ibanez, 2007; Wu et al., 2009; Ascano et al., 2012).

#### Neurotrophin receptor endocytosis

Clathrin-mediated endocytosis upon ligand binding is an important mechanism for both the attenuation and propagation of signals received by many receptor tyrosine kinases (McPherson et al., 2001). A point of difference between the Trk receptors and other receptor tyrosine kinases may be their ability to form long-lived signaling endosomes due to a selective and specific process of clathrin-independent macroendocytosis (Valdez et al., 2005, 2007). These specialized endosomes appear to retain association with markers of early endosomes, and thus fail to mature to late endosomes or progress to lysosomal degradation (Valdez et al., 2005).

In neurons, neurotrophin binding to p75^NTR^ or Trk receptors at the axon terminal induces receptor-mediated endocytosis of the activated receptor complex, and a subset of these internalized receptors are retrogradely transported to the cell body in vesicles that resemble early endosomes (Ehlers et al., 1995; Grimes et al., 1996; Bhattacharyya et al., 1997; Tsui-Pierchala and Ginty, 1999; Watson et al., 1999; Jullien et al., 2002, 2003; Delcroix et al., 2003). These endosomes containing activated Trk receptors can associate with the dynein motor and move along the axon toward the cell body (Bhattacharyya et al., 2002). Data from compartmentalized cultures revealed that NGF applied to the distal axons of sympathetic neurons can support their survival. However, the addition of the specific Trk kinase inhibitor K252a to either the distal axon or the cell body compartment induced cell death suggesting that activated TrkA was required for propagation of the survival signal into the cell and that the downstream effectors of TrkA signaling could not propagate the signal in the absence of activated TrkA and NGF must be retained in the signaling endosomes for the TrkA survival signal to persist (Ye et al., 2003).

### Intracellular localization of internalized Trk receptors

Upon receptor internalization, the exposed cytoplasmic domains of receptor tyrosine kinases can interact with different intermediates found in different subcellular locations, and thereby fine-tune the signaling output of the activated receptors (reviewed by Romanelli and Wood, 2008; Acconcia et al., 2009; Disanza et al., 2009; Sadowski et al., 2009). An example of differential signaling based on altered subcellular localization of activated NGF-TrkA complexes comes from the PC12 cell line that is a useful model of neuronal-like differentiation. PC12 cells express both TrkA and p75^NTR^ and upon serum withdrawal and treatment with NGF, undergo mitotic arrest and extend neurites (Greene and Tischler, 1976; Greene, 1978). It has been demonstrated that in PC12 cells internalization of the activated NGF: TrkA complex is required for the induction of neurite outgrowth but not NGF-mediated survival, suggesting that the intracellular localization of the activated receptors can influence the type of signaling output (Zhang et al., 2000). Activated Trk receptors located at the cell surface promote cell survival signaling via PI3-K/AKT activation whereas Trk receptors signaling from internalized endosomes promote cell differentiation by preferential activation of MEK/ERK pathways (Zhang et al., 2000). A conflicting report using sciatic nerves showed that the p85 subunit of the PI3-K signaling cascade is associated with internal membrane compartments such as early endosomes (Delcroix et al., 2003). It appears that activated Trk receptors are localized to different membrane fractions when comparing PC12 cells and primary neurons (Yano and Chao, 2005), so this may underlie the apparent discrepancy regarding the types of Trk signaling observed in different subcellular locations.

Specific sequences in the cytoplasmic tail of the receptor tyrosine kinases can regulate the targeting of receptors to particular compartments within the cell. In the case of TrkA, a juxtamembrane portion of the receptor between amino acids 473 and 493, that is not present in TrkB or TrkC, is required for NGF-induced recycling of the rat TrkA receptor (Chen et al., 2005). Treatment of primary sympathetic neurons with NGF induced recycling of the TrkA receptor, however application of BDNF induced the degradation of the TrkB receptor. Modification of the TrkB receptor to include this juxtamembrane region from TrkA resulted in the ligand-induced recycling of the modified TrkB receptor.

#### Ubiquitination of Trk receptors

Ubiquitination has also emerged as a very important molecular identifier for the intracellular trafficking of receptor tyrosine kinases including TrkA (Acconcia et al., 2009). Ubiquitin is a polypeptide that can be coupled to proteins via lysine residue modification catalyzed by ubiquitin-activating enzymes, ubiquitin-conjugating enzymes, and ubiquitin ligase enzymes (Komander and Rape, 2012). The different molecular conformations adopted by the different types of ubiquitin labeling can encode various outcomes such as protein degradation, protein activation, altered protein-protein interactions and altered intracellular trafficking (Komander and Rape, 2012). Mono-ubiquitination is defined as the coupling of a single ubiquitin moiety to lysine residues on the target protein. Poly-ubiquitination is the coupling of chains of ubiquitin and the topology of these ubiquitin chains has been thought to dictate proteolytic *versus* non-proteolytic downstream consequences. The internalization of NGF-TrkA has been shown to be regulated by the poly-ubiquitination of TrkA by the ubiquitin ligase TRAF6 in PC12 cells (Geetha et al., 2005) whereas stimulation of PC12 cells and primary cortical neurons with NGF and BDNF has been shown to promote ubiquitination of both TrkA and TrkB, primarily taking the form of multiple sites of mono-ubiquitination rather than poly-ubiquitination (Arevalo et al., 2006). Proteasome inhibition appears to induce neurite outgrowth through TrkA receptor ubiquitination (Song et al., 2009) and recent studies have also highlighted that the proper trafficking of the activated TrkA receptor is essential for NGF-mediated signaling in cultured dorsal root ganglion (DRG) neurons and is regulated by multiple sites of mono-ubiquitination by the ubiquitin ligase Nedd4-2 (Georgieva et al., 2011; Yu et al., 2011). The ubiquitin ligase Cbl has also been implicated in the ligand-dependent ubiquitination of TrkA and subsequent targeting of the receptor to lysosomes for degradation (Takahashi et al., 2011). Clearly ubiquitination and endosomal sorting both have important roles to play in neurotrophin signaling, however, there is still scope for more detailed exploration of the precise molecular mechanisms involved.

Members of the suppressor of cytokine signaling (SOCS) family have recently emerged as ubiquitin ligases regulating expression of a variety of receptors and their signaling pathways. One member of this family in particular, SOCS2, has a variety of functions in the nervous system, many of which overlap with the biological effects of TrkA signaling, suggesting a possible regulatory role for SOCS2 in TrkA function.

## The suppressors of cytokine signaling (SOCS) in the nervous system

The SOCS proteins are a family of intracellular proteins implicated in the negative regulation of a variety of cytokine, growth factor and hormone signals, particularly those mediated by the Janus kinase/signal transducer and activator of transcription (JAK/STAT) signaling pathway (O'Sullivan et al., 2007; Croker et al., 2008; Piessevaux et al., 2008a).

The SOCS proteins have pleiotropic effects in the healthy and diseased nervous system (Wang and Campbell, 2002; Campbell, 2005). They have been implicated in the regulation of diverse cellular processes including neurodevelopment (Turnley et al., 2001; Feng et al., 2007), adult neurogenesis (Ransome and Turnley, 2007), neuroinflammation (Turnley et al., 2002a; Baker et al., 2009; Campbell et al., 2010; Gilli et al., 2010, 2011) and neurotrauma (Stark and Cross, 2006; Qin et al., 2008; Choi et al., 2009; Girolami et al., 2010; Hellstrom et al., 2011). Consistent with the emerging link between excessive inflammation and neurodegenerative disorders (Frank-Cannon et al., 2009), there is now evidence that the SOCS proteins might also have a role to play in the progression of neurodegenerative disorders (Ghosh and Pahan, 2012). In many of the studies cited, the SOCS proteins have been implicated as regulators of inflammatory responses in the central nervous system, however it is important to note that the SOCS proteins also demonstrate neural specific functions in neuronal differentiation. SOCS2 is one member of the SOCS family that is recognized as an important regulator of neuronal function.

### Biochemistry of the SOCS proteins—general

The role of the SOCS proteins has been most extensively studied in the context of cytokine signaling via receptors that lack intrinsic tyrosine kinase activity that recruit effectors such as the cytoplasmic Janus kinases (JAKs) (O'Sullivan et al., 2007). Cytokine binding to the receptor at the cell surface promotes association of the receptor subunits and signals a cascade of downstream phosphorylation events. This signaling pathway begins with the cross-phosphorylation and activation of the receptor-associated JAKs. The JAKs in turn phosphorylate sites on the cytoplasmic tails of the activated receptors thereby creating docking sites for the STAT proteins. Recruitment of these transcription factors is followed by the phosphorylation and dimerization of the STATs. Activated STAT dimers translocate to the nucleus where they can initiate transcription of a variety of genes responsible for survival and proliferation. STAT activation also promotes transcription of negative regulators such as the SOCS family that can suppress further signaling and thus restore sensitivity of the cell to future cytokine stimuli.

**Figure 3:**
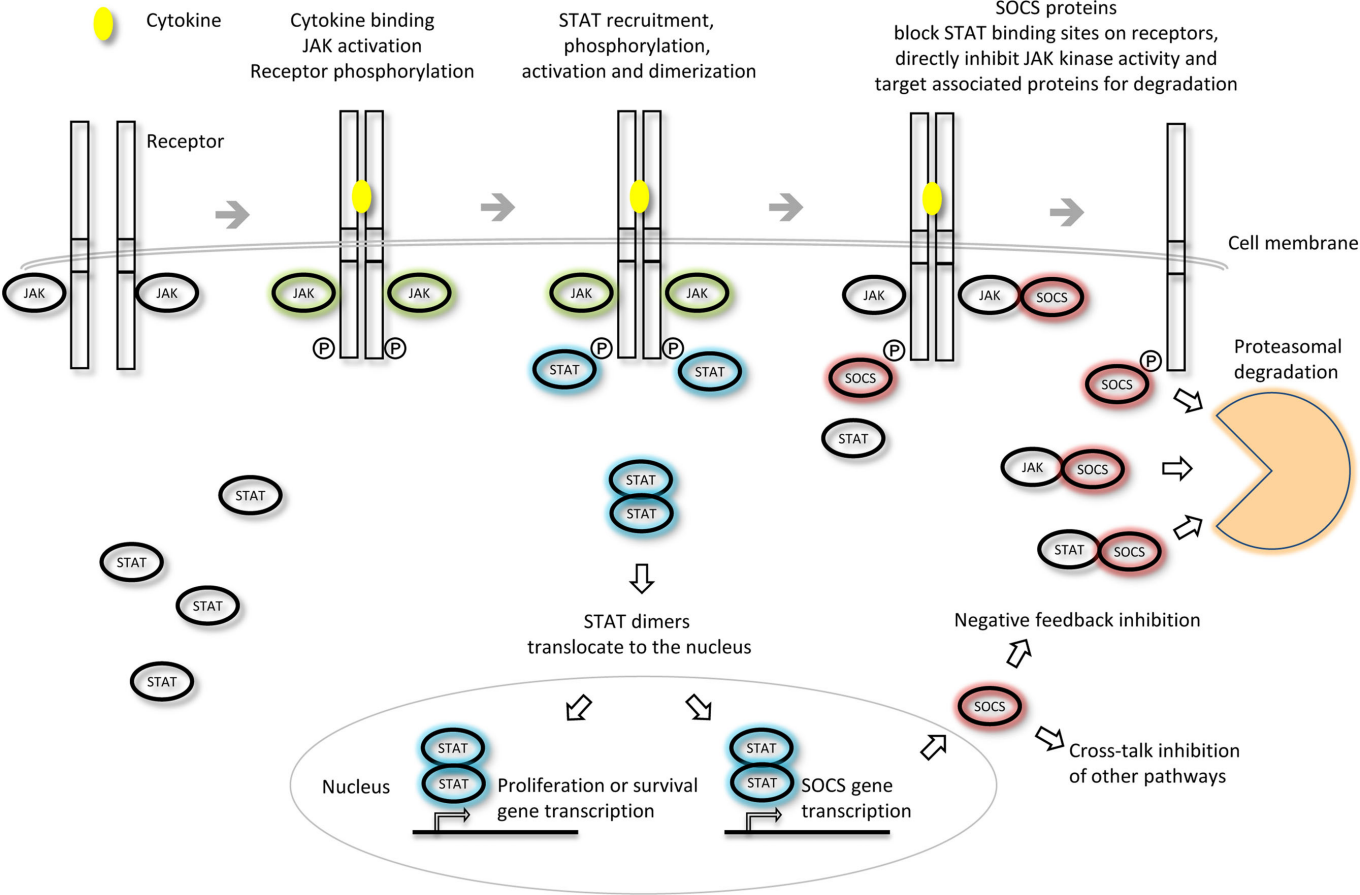
**Inhibition of JAK-STAT cytokine signaling by the SOCS proteins**. The different SOCS proteins can inhibit JAK-STAT signaling via different mechanisms including competition for STAT binding sites on activated receptors, direct inhibition of JAK kinase activity or targeting of associated signaling molecules for proteasomal degradation. Adapted from Palmer and Restifo (2009).

The SOCS proteins were first identified as cytokine-inducible inhibitors of signaling with the characterization of CIS (Yoshimura et al., 1995) and SOCS1 (Endo et al., 1997; Naka et al., 1997; Starr et al., 1997). There are several modes by which the SOCS proteins can inhibit cytokine signaling; competition for STAT binding sites, binding to activated receptors or JAKs and promoting their degradation by the proteasome or direct inhibition of the catalytic site of the JAKs (Figure 3). SOCS proteins can inhibit the downstream effectors of the cytokine stimulus that promoted their initial induction (i.e., “negative feedback”) or inhibit signaling intermediates downstream of independent cytokine stimuli (i.e., “cross-talk”).

**Figure 4:**
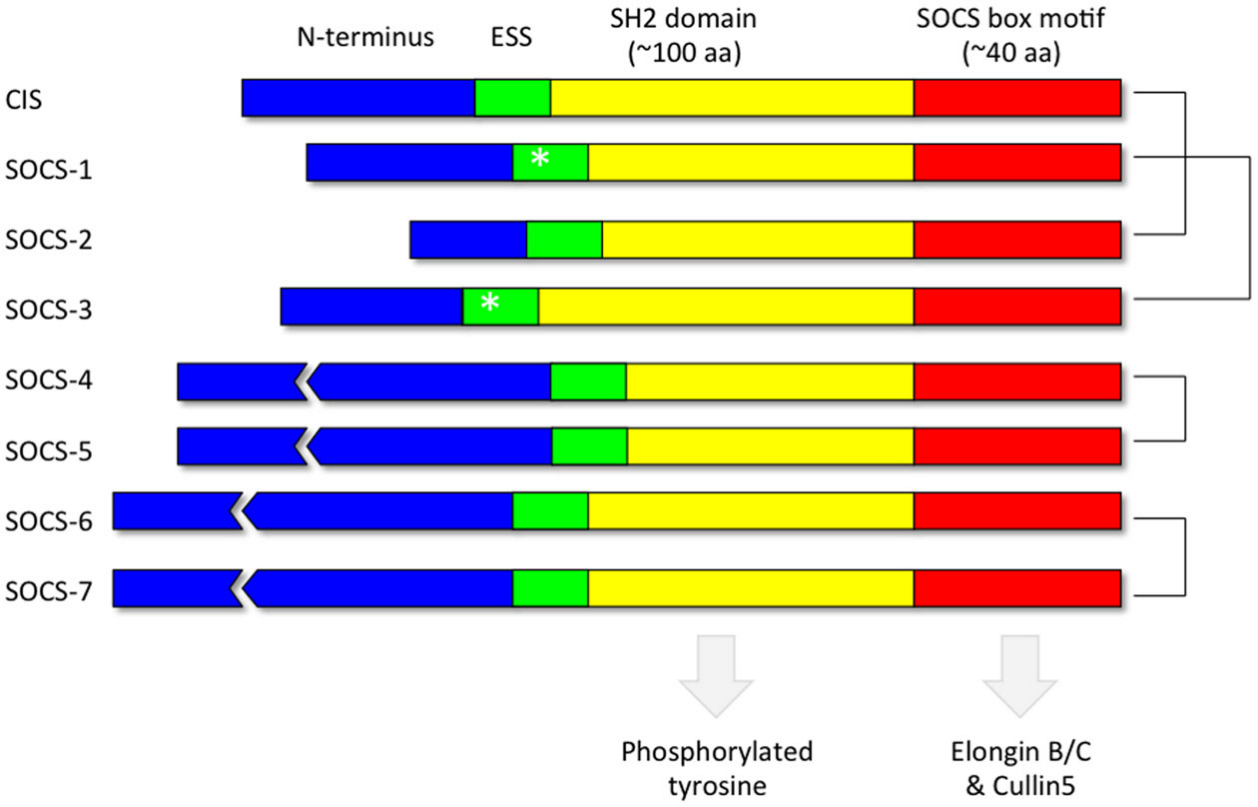
**Domain organization of the SOCS proteins**. Each of the 8 SOCS proteins (SOCS1-7 and CIS) comprise a variable N-terminal region, a central Src Homology 2 (SH2) domain to recognize motifs containing phosphorylated tyrosine residues and a highly conserved C-terminal SOCS box to facilitate association with components of the ubiquitin ligase machinery such as elongin BC and cullin5. The kinase inhibitory region (KIR) (Sasaki et al., 1999; Yasukawa et al., 1999; Kershaw et al., 2013) within the extended SH2 subdomain (ESS) of SOCS1 and SOCS3 are denoted by an asterisk. The most closely related pairs of SOCS proteins according to amino acid sequence similarity are denoted on the right.

Each of the 8 SOCS proteins (SOCS1-7 and CIS) comprise a variable N-terminal region, a central Src Homology 2 (SH2) domain and a highly conserved C-terminal SOCS box (Figure 4). SH2 domains recognize and bind to specific tyrosine phosphorylated motifs within proteins whilst the SOCS box motif interacts with E3 ubiquitin ligases thereby targeting any SOCS-associated proteins for degradation by the proteasome (Kamura et al., 1998; Zhang et al., 1999; Krebs et al., 2002). Pairs of SOCS proteins show varying degrees of similarity in their primary amino acid sequence, suggestive of a degree of functional redundancy amongst members of the family (Hilton et al., 1998). The crystal structures of SOCS2 (Bullock et al., 2006), SOCS3 (Babon et al., 2008) and SOCS4 (Bullock et al., 2007) in complex with elongin BC have been solved. In addition, *in vitro* studies utilizing recombinant purified proteins have demonstrated that the SOCS box motifs of all the SOCS proteins recruit elongin BC before binding Cullin5, an E3 ubiquitin ligase scaffold protein (Babon et al., 2009). A region immediately upstream of the classical SH2 domain, known as the extended SH2 subdomain (ESS), appears crucial to the formation of a stable interface between the SOCS box and the SH2 domain in a subset of the SOCS family comprising CIS, SOCS1, SOCS2, and SOCS3 (Bullock et al., 2006).

The SOCS proteins couple specific phosphotyrosine-motif recognition with ubiquitin-ligase activity and are thus potent inhibitors of a variety of cytokine signaling pathways. As previously mentioned, the SOCS proteins can participate in “negative feedback” inhibition or “cross-talk” suppression of independent cytokine stimuli. This cross-talk has been shown to extend to the cross regulation amongst the SOCS proteins. SOCS2, SOCS6, and SOCS7 appear to form a subgroup of the SOCS family that have been shown to interact with the SOCS box of all members of the SOCS family and may be required to regulate the stability of other SOCS proteins (Piessevaux et al., 2006). This cross regulation might enable late-induction SOCS proteins (such as SOCS2) to down regulate early-induction SOCS proteins (such as CIS, SOCS1, and SOCS3) so the cell can restore sensitivity to future cytokine stimulation (Tannahill et al., 2005; Piessevaux et al., 2006).

#### The regulation of receptor trafficking by the SOCS proteins

The SOCS proteins typically act by regulating the ubiquitin tagging of signaling intermediates to promote their proteasome-dependent degradation and thus suppress further signaling. As previously mentioned, a more complex role for post-translational modifications such as ubiquitination has emerged whereby the type and extent of ubiquitin tagging can also dictate the sorting of membrane-associated proteins to early endosomes which can continue to signal after internalization. It is intriguing to speculate the important role that ubiquitin-ligases such as the SOCS proteins may play in both determining the strength or duration of signaling events, and fine-tuning the type of responses of cells such as neurons to extracellular cytokine stimuli. One example that implicates the SOCS proteins in this receptor trafficking role has been observed in the SOCS3 dependent transport of the granulocyte colony stimulating factor receptor (G-CSFR) (Irandoust et al., 2007). G-CSFR transport from the early endosomes to the lysosomes was impaired when a membrane-proximal lysine residue of the G-CSFR was mutated to prevent ubiquitin conjugation at this site and altered ubiquitin conjugation of this receptor resulted in altered intracellular signaling outcomes.

### The biochemistry of SOCS2

SOCS2 was isolated in a yeast-two-hybrid screen in which the cytoplasmic domain of the insulin-like growth factor-1 (IGF-1) receptor was used as bait to screen a cDNA library of proteins expressed in the human fetal brain (Dey et al., 1998). IGF-1 is a second messenger of GH and the essential role of SOCS2 in the negative regulation of the GH/IGF-1 pathway was subsequently validated *in vivo* by the phenotype of the SOCS2KO mice (Metcalf et al., 2000). The activated GHR engages the JAK/STAT pathway and accordingly the observed induction of SOCS2 by GH (Adams et al., 1998; Tollet-Egnell et al., 1999) is supported by the presence of putative STAT5 transcription factor binding sites, amongst others, in the vicinity of the *Socs2* gene (Laz et al., 2009).

The transfection of 293T cells with a series of SOCS2 deletion constructs in addition to the GH receptor highlighted the structural motifs required for the SOCS2 downregulation of signaling via the GH receptor (Greenhalgh et al., 2005). Removal of the SOCS2 N-terminus relieved inhibition of GH signaling and point mutation of critically conserved residues within the phosphotyrosine-binding pocket of the SH2 domain produced partial or complete loss of inhibitory function. Furthermore, deletion of the SOCS box relieved inhibition of GH signaling and actually resulted in enhanced signaling even at low concentrations. The inhibitory role of SOCS2 has been shown to rely on the C-terminal SOCS box in the case of proteasomal degradation of the phosphorylated proline-rich tyrosine kinase 2 (Pyk2) (Lee et al., 2010). The most recent development in our understanding of SOCS2 biochemistry came with the demonstration that SOCS2 can directly regulate the levels of the growth hormone receptor in a 293T overexpression system (Vesterlund et al., 2011). The SOCS2 ubiquitin ligase complex was shown to directly ubiquitinate the GH receptor and co-expression of SOCS2 decreased the half-life of the GHR. This effect of SOCS2 overexpression was removed by inhibition of the proteasome and mutation of GHR at Y487 and an intact SOCS box was required for the stabilization of the SOCS2-ubiquitin ligase complex.

Overexpression of SOCS2, both *in vivo* and *in vitro*, has unveiled a complex role for SOCS2 in the modulation of GH signaling and it is clear that SOCS2 can both inhibit and enhance GH signaling (Favre et al., 1999; Greenhalgh et al., 2002a). There are several explanations for the enhancement of GH signaling observed when SOCS2 is overexpressed. It is possible that the overexpressed FLAG-SOCS2 used in the *in vitro* studies is non-functional and inhibiting the role of endogenous SOCS2, thus the overexpressed SOCS2 is acting as a dominant-negative mutant (Greenhalgh et al., 2002a). Alternatively, at high concentrations SOCS2 might be competing for SOCS3 binding sites on the GH receptor (residues Y332 and Y487) and since SOCS3 is considered a more potent inhibitor of GH signaling, displacing SOCS3 from these binding sites might indirectly enhance GH signaling (Greenhalgh et al., 2002a). Other groups (Tannahill et al., 2005; Piessevaux et al., 2008a) propose that since SOCS2 can bind all members of the SOCS family, SOCS2 may accelerate the turnover of other SOCS proteins via a “cross-regulatory mechanism” that would allow SOCS2 to enhance cytokine responses by accelerating proteasome-dependent turnover of SOCS3. In addition, binding to elongin BC has been shown to be essential for the proper folding and substrate recognition by the SOCS protein CIS (Piessevaux et al., 2008b). If the pool of available elongin BC were depleted by binding to overexpressed SOCS2, this may indirectly enhance cytokine responses that would normally be suppressed by other SOCS proteins.

### SOCS2 and mouse biology—general

SOCS2 was initially characterized as a negative regulator of growth hormone (GH) signaling (Metcalf et al., 2000). The SOCS2 mediated downregulation of GH signaling is best demonstrated by the gigantism phenotype of mice lacking SOCS2 (Metcalf et al., 2000). The SOCS2KO mice were approximately 40% heavier by 6 weeks of age and this corresponded to an increase in bone length and the size of most organs, although no hematological abnormalities were observed. The difference in body size was accelerated at about 3–4 weeks of age, and this was noted as the time point at which growth hormone receptor is upregulated in many tissues (Shoba et al., 1999). Other aspects of the SOCS2KO phenotype that suggested a role for SOCS2 in GH signaling included an increase in the levels of the GH second messenger insulin-like growth factor-1 (IGF-1), decreased levels of major urinary protein and thickening of the skin due to collagen accumulation.

Mice lacking both STAT5b and SOCS2 and were found to grow normally (Greenhalgh et al., 2002b) which suggested that the SOCS2 modulation of JAK/STAT signaling downstream of the GH receptor depends on the activity of STAT5b. Accordingly, SOCS2 has been shown to interact with the GH receptor *in vivo* and with STAT5b binding sites on the cytoplasmic tail of the GH receptor (Y487 and Y495) *in vitro* (Greenhalgh et al., 2005). The importance of SOCS2 as a negative regulator of GH signaling *in vivo* was further exemplified by the absence of the SOCS2KO phenotype when endogenous GH was also removed (Greenhalgh et al., 2005).

Given that the SOCS2KO mice were hyper-responsive to GH signaling, it was presumed that increased SOCS2 expression would yield mice hypo-responsive to GH signaling with a phenotype similar to the dwarfism observed in GH receptor knockout mice (Zhou et al., 1997). On the contrary, transgenic mice that overexpress SOCS2 (SOCS2TG) displayed a modest overgrowth phenotype indicating that when SOCS2 is either absent or expressed at high levels there is enhanced signaling via the GH receptor (Greenhalgh et al., 2002a). Whilst the SOCS2TG mice were approximately 15% heavier at 3 weeks of age, they did not display any other abnormalities with respect to specific tissues or hematopoietic profile.

### SOCS2 and neurobiology

#### Neuronal expression profile of SOCS2; probable roles in neuronal proliferation and differentiation

SOCS2 is notable for the high levels of RNA expression in neurons of the developing and adult mouse brain, and interestingly the onset of SOCS2 expression appears to coincide with that of neuronal differentiation (Polizzotto et al., 2000). The neural specific expression of SOCS2 during development prompted investigation into the role of SOCS2 in neuronal differentiation. SOCS2 is expressed in neural stem cells and neurons most highly at E14 which is a time point that coincides with a peak in neuronal generation (Polizzotto et al., 2000). More detailed *in vitro* analysis of the neuronal expression profile of SOCS2 revealed that it is expressed in cultured embryonic day 10 (E10) neuroepithelial cells and E17 cortical neurons but not cortical astrocytes or adult multipotent stem cells (Turnley et al., 2002b). In the neuroepithelial cultures, *Socs2* gene expression was increased above basal levels by the addition of LIF, interferon gamma, OsM and CNTF, and LIF promoted *Socs2* gene expression in E17 cortical neuron cultures (Turnley et al., 2002b). Notably, growth hormone (GH) did not influence *Socs2* expression in either cell type (Turnley et al., 2002b) unlike previous studies in which GH induced expression of several SOCS proteins including CIS, SOCS1, SOCS2 and SOCS3 in non-neuronal cell lines and tissues (Adams et al., 1998; Tollet-Egnell et al., 1999). A link between SOCS2 and the transcriptional regulation of neurogenesis has been found in the GH regulation of the proneurogenic basic helix loop helix transcription factor Neurogenin-1 (Turnley et al., 2002b).

#### Neuroanatomical consequences of SOCS2 overexpression

Mice with altered growth hormone responsiveness, including SOCS2KO (hyper-responsive), SOCS2TG (hypo-responsive) and GH receptor null (GHRKO) (non-responsive) mice, exhibit a variety of alterations in neural architecture (Ransome et al., 2004; Ransome and Turnley, 2005). The changes in neural architecture observed in SOCS2TG and SOCS2KO mice implicate this gene in neuronal differentiation and neurite outgrowth. Interestingly, despite the fact that loss of SOCS2 has an effect on mouse body and organ size (Metcalf et al., 2000), it has no effect on brain size, although there was an effect on neuronal density and composition (Ransome et al., 2004; Ransome and Turnley, 2005). The SOCS2KO mice have decreased cortical neuron density whereas the SOCS2TG and GHRKO mice exhibit an increased cortical neuron density. The SOCS2TG mice have increased synaptic density and dendritic branching in the cortex, in contrast to the sparse dendritic branching of pyramidal neurons in the cortex of the SOCS2KO and GHRKO mice. Further, SOCS2TG mice and GHRKO mice had markedly increased numbers of Calretinin and Calbindin positive cortical interneurons, although expression of other markers, such as Parvalbumin and somatostatin were not significantly altered. Furthermore, the overall number of gamma-aminobutyric acid positive interneurons did not appear to change between genotypes. The altered expression of cortical interneuron markers observed in SOCS2TG and SOCS2KO mice suggests that SOCS2 may regulate expression of specific transcription factors required for interneuron subtype specification or that expression of the interneuron markers might be modulated by the amount of synaptic activity within a cell and thus the altered levels of expression in the SOCS2TG and SOCS2KO mice might simply arise from changes in neuronal circuitry and synaptic activity (Ransome and Turnley, 2005). Juxtaposition of the neuroanatomical phenotypes of mice with altered responsiveness to GH signaling (SOCS2KO hyper-responsive > SOCS2TG hypo-responsive > GHRKO non-responsive) reveals that whilst cortical neuron density correlates with increasing responsiveness to GH, the measures of dendritic branching or some interneuron populations do not. This invites speculation that SOCS2 can also modulate other signaling events independent of GH signaling in neural cells.

#### SOCS2 and neurite outgrowth

The proper formation of connections between cells of the nervous system is critically dependent upon the extension of processes or “neurites” at the surface of neurons. As mentioned previously, a useful model of neuronal-like differentiation is the PC12 cell line derived from a rat pheochromocytoma that undergoes mitotic arrest and extends neurites when serum is withdrawn and NGF is applied (Greene and Tischler, 1976; Greene, 1978). Recent evidence suggests that SOCS2 may play a role in neurite initiation as well as regulating neurite length. Overexpression of SOCS2 in PC12 cells induces neurite outgrowth under non-differentiating conditions (Goldshmit et al., 2004a) and primary cortical neurons derived from transgenic mice that overexpress SOCS2 demonstrate an increase in neurite length and neurite number (Goldshmit et al., 2004b). Despite the fact that SOCS2 is required to overcome the inhibitory effects of GH on neuronal differentiation, SOCS2 has also been shown to enhance neurite outgrowth in neurons derived from neural progenitor cells and this activity was not inhibited by the addition of GH (Scott et al., 2006). These data suggest that the mechanisms by which SOCS2 regulates neuronal differentiation and neurite outgrowth may be independent.

Since these initial reports of SOCS2 involvement in neurite outgrowth, there have been other studies implicating various members of the SOCS family in neurite outgrowth including SOCS3 and SOCS6 (Miao et al., 2006; Gupta et al., 2011). SOCS7 may also emerge as an important regulator of neurite outgrowth as it has been identified as an Nck interacting protein (Kremer et al., 2007) and overexpression of Nck in the PC12 cell line has been shown to enhance proliferation and block NGF and bFGF induced differentiation by a MAPK independent mechanism (Rockow et al., 1996).

## SOCS2 as a novel regulator of TrkA localization and signaling

While SOCS2 has broad ranging effects on neuronal differentiation, maturation and survival, the molecular basis for SOCS2 activity in neurons is still largely unresolved. An altered responsiveness to GH does not appear to address all the changes observed in the neural architecture of SOCS2KO and SOCS2TG mice or the enhanced neurite outgrowth in neurons derived from neural progenitor cells. Previous studies showed that overexpression of SOCS2 in PC12 cells resulted in enhanced neurite outgrowth, and this response was enhanced by the addition of NGF (Goldshmit et al., 2004a). Thus, the possibility that SOCS2 might be regulating the neuronal response to NGF prompted further investigation.

Neonatal dorsal root ganglia (DRG) contain a large population of TrkA+ neurons that require NGF for survival and promotion of neurite outgrowth. Analysis of DRG neuron cultures derived from SOCS2TG and SOCS2KO mice showed that increased or no expression SOCS2 respectively, regulated NGF-mediated neurite outgrowth, with SOCS2 overexpression increasing and lack of SOCS2 decreasing neurite length and complexity (Uren et al., 2014). Surprisingly however, SOCS2 overexpression did not influence NGF-induced survival of DRG neurons, suggesting that SOCS2 differentially regulates TrkA-related signal transduction pathways. In order to try to dissect the mechanisms by which this differential regulation occurred, a range of binding studies, expression and signal transduction analyses were performed using SOCS2 and TrkA transfected 293T cells (contain no endogenous TrkA), SOCS2 transfected PC12 cells (contain endogenous TrkA and SOCS2) and SOCS2TG and wildtype DRG neurons (contain endogenous TrkA and SOCS2).

Co-immunoprecipitation of SOCS2 and TrkA mutant proteins in 293T cells showed that the juxtamembrane region of TrkA was required for binding of SOCS2, with possible involvement of the kinase domain as well. Overexpression of SOCS2 increased total TrkA levels in transfected 293T cells and PC12 cells and increased cell surface expression of TrkA in PC12 cells and SOCS2TG DRG neurons (Uren et al., 2014), suggesting that SOCS2 regulates TrkA protein turnover and cellular localization. Further, SOCS2 overexpression increased the extent and duration of NGF-induced activation of signal transduction pathways involved in neurite outgrowth such as ERK1/2 and pAKT (Uren et al., 2014). These effects of SOCS2 on NGF mediated neurite outgrowth but not survival, with altered TrkA surface expression and enhanced signal transduction pathways are summarized in Table 1.

**Table 1:** **SOCS2:TrkA interaction and outcome summary**.

	**Outcome of increased SOCS2 expression**	
	**No NGF**	**With NGF**
Survival	No change (DRG)	No change (DRG)
Neurite outgrowth	Increased ↑ (PC12)	Increased ↑↑ (PC12 and DRG)
TrkA: total protein levels	Increased ↑ (PC12 and 293T)	Increased ↑ (PC12 and 293T)
TrkA: surface protein levels	Increased ↑ (PC12 and DRG)	Increased ↑ (PC12) No change (DRG)
Signaling: pTrkA, pAKT, pERK1/2	pERK1/2 Increased ↑ Others no change (PC12)	Increased ↑ (PC12)

*Increased expression of SOCS2 (relative to endogenous levels of SOCS2), either in SOCS2TG mice (DRG neurons) or by transfection of cell lines (PC12 and 293T cells) alters the outcome of NGF-induced signaling. Increased SOCS2 does not affect NGF-mediated DRG neuron survival, with equivalent levels of survival with NGF and cell death in the absence of NGF. However, increased SOCS2 levels promote increased neurite outgrowth under basal conditions, which is further enhanced by NGF. It also increases total and surface TrkA levels (in PC12 cells the increased surface levels correlate with increased total levels) and increases the level and duration of signaling via the PI-3 kinase (AKT) and ERK1/2 pathways. The mechanism for this differential outcome of SOCS2 on NGF-mediated survival vs. neurite outgrowth remains to be determined, as does how SOCS2 influences neurite outgrowth, but likely involves ubiquitination and altered TrkA cellular localization due to the E3 ubiquitin ligase activity of the SOCS box*.

While the mechanism by which SOCS2 regulates TrkA signaling and functional outcome remains to be determined, it most likely revolves around altered TrkA localization induced by SOCS2-mediated ubiquitination. This could regulate the amount of TrkA receptor at the cell surface and influence the rate at which it is internalized, recycled, or transported to different intracellular compartments, thereby modulating sensitivity of a cell to NGF and altering downstream signal transduction pathways. Ubiquitination of the lysine in the TrkA juxtamembrane KFG motif regulates TrkA expression levels and localization, with deletion of this domain leading to decreased ubiquitination, increased TrkA levels and increased phosphorylation of the AKT and MAP kinase pathways (Kiris et al., 2014), mirroring the effects of SOCS2 overexpression (Uren et al., 2014). While SOCS2 was able to bind to TrkA with the KFG domain deleted (TrkAΔ450–452) further functional effects of this deletion on TrkA expression, localization or functional effects such as neurite outgrowth were not examined (Uren et al., 2014). However, the similarity between findings when SOCS2 is overexpressed or the TrkA KFG domain is deleted suggests that regulation of ubiquitination by SOCS2, either directly or indirectly, at the KFG motif may be at least part of the mechanism regulating TrkA localization, expression levels and downstream signal transduction. Interestingly, the converse effects of decreased surface TrkA levels have also been described. Depletion of NHE5 [a Na(+)/H(+) exchanger that acidifies recycling endosomes to promote cell surface expression] resulted in decreased levels of TrkA at the cell surface in PC12 cells, with concomitant decreased phosphorylation of AKT and ERK1/2 and decreased neurite outgrowth in response to NGF treatment (Diering et al., 2013).

## Conclusions

Neurotrophins and their receptors are expressed in specific areas throughout the nervous system. Their expression levels, signal transduction pathways and localization are tightly regulated by a range of different mechanisms, including dephosphorylation and degradation. SOCS proteins are best known for their role as negative feedback inhibitors of cytokine signaling but have been shown to have a range of other roles, depending on cell type and receptor under study. Their structure, which includes an SH2 interaction domain and the SOCS box ubiquitin ligase interaction domain, suggests that they may interact with many more proteins. This certainly appears to be the case for the TrkA receptor and SOCS2. However, given that SOCS2 has also been shown to bind to the BDNF and NT-3 neurotrophin receptors (Uren et al., 2014), it is possible that SOCS2 plays even broader roles in regulation of neurotrophin signaling. Further, such crosstalk is not necessarily confined to the nervous system and may play a role in hematopoiesis, given the expression and function of Trk receptors in early hematopoietic cells (Chevalier et al., 1994; Ip, 1998; Li et al., 2009; Rezaee et al., 2010). While the mechanisms remain to be determined, SOCS2 and perhaps other SOCS molecules may provide new therapeutic targets for regulation of pathological signaling via neurotrophin receptors and suggests that further research into the field is warranted.

### Conflict of interest statement

The authors declare that the research was conducted in the absence of any commercial or financial relationships that could be construed as a potential conflict of interest.
